# Early diagnosis of liver failure in septic patients using the maximal liver function capacity test (LiMAx test): comparison with conventional methods

**DOI:** 10.1186/cc12341

**Published:** 2013-03-19

**Authors:** M Kaffarnik, M Stockmann, J Lock

**Affiliations:** 1 Charite Berlin Campus Virchow, Berlin, Germany

## Introduction

Patients with bacterial sepsis often suffer from multiorgan failure. No reliable parameter exists for the exact estimation of the liver function. The indocyaningreen test (ICG test) showed a correlation between liver function and mortality rate. The new maximal liver function capacity test (LiMAx test) is a real-time method to investigate the liver function.

## Methods

Thirty septic patients were prospectively included in the study. The LiMAx test was performed on days 0, 2, 5 and 10 after sepsis onset and was compared with ICG test and liver-specific laboratory parameters. Primary endpoint was the mortality rate after 90 days. Secondary endpoint was the comparison with the ICG test.

## Results

**Figure 1:**
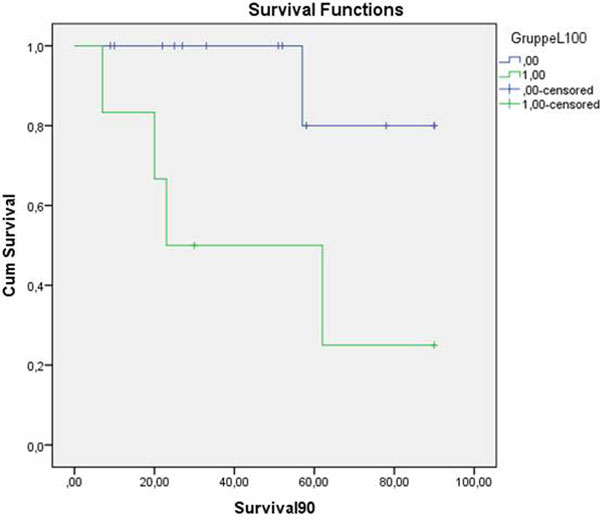
**Kaplan-Meier survival curve of patients with high and low LiMAx results**.

The LiMAx test showed low results initially with increasing values on days 5 and 10. The 90-day mortality rate in patients with a low LiMAx test on day 2 (<100 μg/kg/hour) was significantly higher than in patients with a LiMAx test >100 μg/kg/hour. The LiMAx test was comparable with the ICG test. Patients with indication for renal replacement therapy during hospital treatment showed significantly lower LiMAx values than patients without dialysis. See Figure 1.

## Conclusion

With the LiMAx test we can detect a liver dysfunction in septic patients early on days 0 to 2. The LiMAx test is equal with the ICG test and a result of <100 μg/kg/hour on day 2 suggests a low probability of survival in septic patients.
